# TRIM66-HP1γ remodels the chromatin through phase separation

**DOI:** 10.52601/bpr.2024.240038

**Published:** 2025-02-28

**Authors:** Siyuan Shen, Feng Chen, Yifan Zhang, Fudong Li, Xuebiao Yao, Dan Liu, Yunyu Shi, Liang Zhang

**Affiliations:** 1 Center for Advanced Interdisciplinary Science and Biomedicine of IHM, Division of Life Sciences and Medicine, University of Science and Technology of China, Hefei 230027, China; 2 Ministry of Education Key Laboratory for Membraneless Organelles and Cellular Dynamics, University of Science and Technology of China, Hefei 230027, China; 3 Hefei National Research Center for Cross disciplinary Science, University of Science and Technology of China, Hefei 230027, China

**Keywords:** Chromatin, TRIM66-HP1γ, Facultative heterochromatin, Phase separation

## Abstract

Chromatin contains not only heterochromatin (HC) and euchromatins (EC) but also facultative heterochromatin (fHC), which experience the dynamic remodeling between HCs and ECs by different regulators. The regulation of fHCs involves lots of different cell functions, like genomic stability and gene transcription. Heterochromatin protein 1 (HP1) recognizes methylated H3K9 and reshapes the chromatin into the fHCs through liquid–liquid phase separation (LLPS). Among the three members of the HP1 family, HP1α can condensate by itself and HP1β forms granules with the help of TRIM28, while the HP1γ cannot phase separation alone either and the coordinator is still unclear. So, in this study, we investigated the molecular mechanism of how HP1γ interacts with TRIM66 through PxVxL motif. Based on that, we examined the key regions that controlled the TRIM66-HP1γ co-phase separation behaviors both *in vitro* and *in vivo*. Furthermore, we proved that the liquid granules of TRIM66-HP1γ and chromatin highly correlated with H3K9me3 sites, which indicated the relationship with DNA damage response. Finally, combined with our previous study, we proposed the system for how TRIM66 remodeled the chromatin into compressed fHC through the TRIM66-HP1γ-H3K9me3 axis with liquid–liquid phase separation.

## INTRODUCTION

In eukaryotic cells, the genetic material is organized into chromatin. Chromatin is the complex assemblage of DNA, histone proteins, and other non-histone protein components (Jenuwein and Allis 2001). The chromatins wrap into the chromosomes during the cell division and loosen back to form chromatins after mitosis (Reyes *et al.* 2021; Stephens *et al.* 2019; Zheng and Xie 2019). The chromatins are separated into condensed and decondensed chromatin states based on the compressing status. In 1928, Emil Heitz first defined heterochromatin (HC) and euchromatin (EC) based on genetically dormant and active, respectively (Heitz 1928). A major conceptual advance in recognizing the dynamic nature of HC has been reported through extensive studies of transposable elements in plants by Barbara McClintock (Feschotte *et al.* 2002). Subsequent improvements in staining methods and the development of electron microscopy showed that HC could be subdivided into constitutive and facultative HC (Machida *et al.* 2018; Trojer and Reinberg 2007). Constitutive heterochromatin (cHC) is majorly located at the centromere and telomere regions. Facultative heterochromatin (fHC) then designates genomic regions in the nucleus of a eukaryotic cell that has the opportunity to adopt open or compact conformations within temporal and spatial contexts (Trojer and Reinberg 2007). fHC experiences the interconversion between HC and EC to regulate the genome transcription and repression. The molecular mechanisms underlying the establishment and maintenance of fHC are involved in multiple factors, which include the incorporation of specific or alternative chromatin components; histone posttranscriptional modifications (PTMs); concerted action of trans-acting factors, and subnuclear localization (McCarthy *et al.* 2023; Sanulli *et al.* 2019; Singh and Newman 2022).

Heterochromatin protein 1 (HP1) is a key element in heterochromatin formation. Heterochromatin is characterized by methylation of histone H3K9, which is recognized by HP1 (Faivre and Schubert 2023; Sharda and Humphrey 2022; Wang *et al.* 2019). HP1-family proteins possess an N-terminal chromodomain (CD) that acts as a specific reader for H3K9me2 or H3K9me3 (Eissenberg and Reuter 2009). A chromo shadow domain (CSD) at the C-terminal region promotes homodimerization and provides a surface for ligand interaction and a flexible hinge region connecting the CD and CSD domains (Azzaz *et al.* 2014; Elgin and Reuter 2013; Liu *et al.* 2017). Humans possess three main HP1 paralogs — alpha (α), beta (β), and gamma (γ) — encoded by the *CBX5*, *CBX1*, and *CBX3* genes, respectively (Sanulli *et al.* 2019).

Irene *et al.* reported that HP1a, a *Drosophila* homolog of HP1, regulated the heterochromatin formation and promoted the genome stability in *Drosophila* in 2011 (Chiolo *et al.* 2011). In 2017, Strom *et al.* also analyzed heterochromatin formation in the nuclei of early-stage *Drosophila* embryos, and they observed that heterochromatin domains labeled with HP1a bear the hallmarks of phase-separated compartments (Strom *et al.* 2017). At the same time, Larson *et al*. compared the properties of the three human forms of HP1 *in vitro* (Larson *et al.* 2017). They discovered that one member among them, HP1α, can spontaneously phase-separate in solution and form liquid-like droplets. As commented in the nature news and views, these two studies provided a fresh perspective on how heterochromatic domains perform their function (Klosin and Hyman 2017). It seems that the access of molecules to heterochromatin might be determined by selectivity at the interface between the liquid drop and its surroundings rather than by simple spatial confinement in the dense chromatin meshwork (May *et al.* 2022). The dynamic properties of a liquid-like domain might facilitate the repair of DNA breaks within heterochromatin. Liquidity would allow relocation of the damaged DNA segment from the center to the boundary, where it can be accessed by the large protein complexes required for repair. Complexes that mediate transcription or replication could also be rejected from the damaged heterochromatic regions, allowing them to act at the appropriate times without disrupting the domain as a whole (Morrison and Thakur 2021).

Here, we just provide another example that is the interaction between TRIM66 and HP1γ promotes the liquid-liquid phase separation (LLPS) and drives facultative heterochromatin domain formation, and this may relevant to TRIM66 function of DNA damage repair in embryonic stem cells.

**Figure 1:**
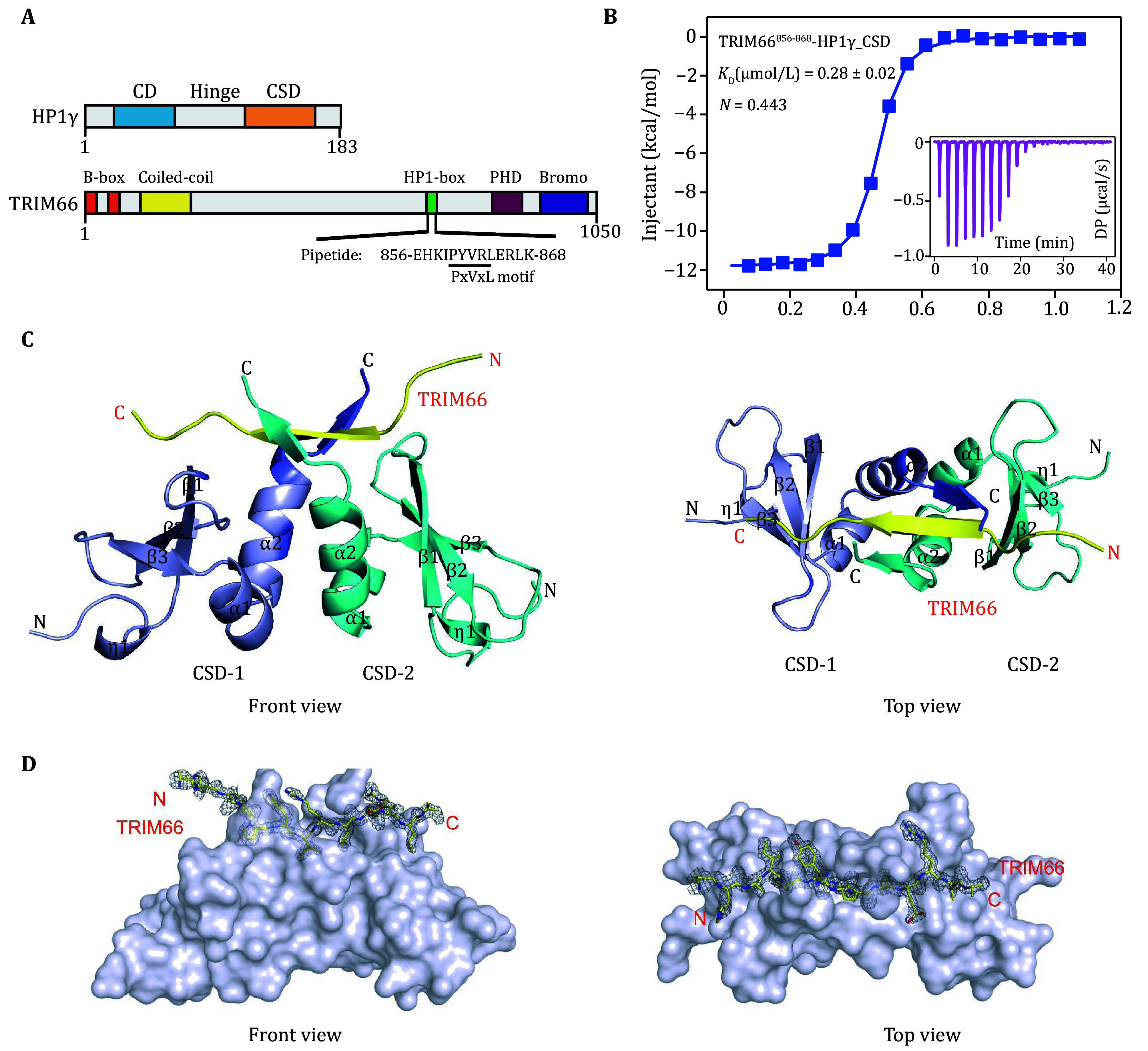
HP1γ chromo shadow domain (CSD) interacts with the TRIM66 PxVxL motif peptide. **A** The domain architectures of human HP1γ and TRIM66. **B** The ITC fitting results of HP1γ-CSD with the TRIM66 PxVxL motif peptide. **C** Cartoon representation of HP1γ-CSD dimer (cyan and purple) in complex with the peptide (yellow) (front view and top view). **D** The surface views of HP1γ-CSD and TRIM66^856-868^ complex (front view and top view). The surface of the HP1γ-CSD is colored grey, while the TRIM66^856-868^ peptide is represented as a yellow ribbon. The 2*F*o-*F*c omit density map of the TRIM66 PxVxL motif peptide was contoured at 1.0 σ

TRIM family proteins are characterized by the tripartite motifs that are involved in the regulation of the post-translational modification of a variety of proteins. They are key mediators of mRNA degradation and epigenetic modifications and play a crucial role in regulating cell transcriptional and translational activities. TRIM proteins are divided into several sub-families (Class I to Class XI) (Hatakeyama 2011). TRIM66, together with TRIM24, TRIM28, and TRIM33, belongs to one subfamily and is called the TIF1 family (transcriptional intermediary factor 1) (Khetchoumian *et al.* 2004). Four of the TIFs family proteins, TRIM24 (TIF1α), TRIM28 (TIF1β), TRIM33 (TIF1γ) and TRIM66 (TIF1δ), share similar protein architectures: N terminus is composed of two B-boxes and coiled-coiled domains while C-terminal region contains plant homeodomain (PHD)-Bromodomains. The biggest difference between TRIM66 and the other members in the TIFs family is TRIM66 doesn’t have a ring domain in the N-terminal domain (Fig. 1A). In addition, all three TIFs family proteins (except TRIM33) contain a PxVxL motif which is necessary and sufficient for HP1 binding (Khetchoumian *et al.* 2004). A previous study in our laboratory reported that the TRIM66 recognized the unmodified H3R2-H3K4 and acetylated H3K56 through the PHD-Bromo domains and responded to the DNA damage repair in embryonic stem cells (mESCs) (Chen *et al.* 2019). The PxVxL motif, which is near the PHD domain in TRIM66, can be recognized by the HP1γ CSD domain more preferable than the CSDs of HP1α and HP1β (Khetchoumian *et al.* 2004). Unlike HP1α, HP1β and HP1γ can’t spontaneously phase-separate in solution and form liquid-like droplets. HP1β and HP1γ need other regulators’ help to form the condensation and control the organization of fHC around the marked histone sites. Pilong Li has reported that the multivalent interactions among TRIM28, HP1β and SUV39H1 can promote phase separation to form macromolecule-enriched liquid droplets and drive chromatin compartmentalization and fHC formation (Wang *et al.* 2019). As either HP1α itself or HP1β-TRIM28 can form condensation together with modified histone to reshape the chromatin, we proposed that the interaction among TRIM66, HP1γ and the nucleosome may also promote the formation of the liquid-like droplets and regulate the formation of facultative heterochromatin.

In this study, we determined the crystal structure of the chromo shadow domain (CSD) of HP1γ in complex with PxVxL peptide derived from TRIM66 and identified the key residues that respond to the interaction between them. Then we discovered that TRIM66 and HP1γ can phase separated with nucleosomes *in vivo* and *in vitro*. Based on these results, we mapped the N terminal and B-box-B-Box-Coil-Coil domains (BBCC) as the key regulators to mediate the condensation of H3K9me3 labeled chromatin regions. Combining all the results, we finally demonstrated the molecular basis of how TRIM66 silenced the H3K9me3 through LLPS by the TRIM66-HP1γ-H3K9me3 histone axis.

## RESULT

### Crystal structure of the HP1γ-CSD and TRIM66 PxVxL motif complex

Previous studies have revealed that HP1γ mainly utilizes its C-terminal CSD to interact with TRIM66 protein (Khetchoumian *et al.* 2004) (Fig. 1A). To understand the molecular structure of the HP1γ-CSD complexes with TRIM66, we cloned, expressed and purified HP1γ-CSD protein and synthesized the TRIM66^856–868^ peptide, which includes PxVxL motif. Then we performed isothermal titration calorimetry (ITC) assays to determine the binding affinity between HP1γ-CSD and TRIM66^856–868^ peptide. The result showed that HP1γ-CSD binds TRIM66^856–868^ with a dissociation constant (*K*_D_) of about 0.28 μmol/L (Fig. 1B and supplementary Table S1). In combination with the N value of the ITC results, HP1γ-CSD and TRIM66^856–868^ formed a stable 2:1 stoichiometry complex consistent with previous reports.

Next, we crystalized the HP1γ-CSD-TRIM66^856–868^ complex protein and successfully resolved the crystal structure of the complex at 1.8 Å resolution (supplementary Fig. S1A and Table S2). The space group of the crystal structure is *P*2_1_, while each crystallographic asymmetric unit contains two HP1γ-CSD dimers and two TRIM66^856–868^ peptides. Consistently with the 2:1 stoichiometry model based on the results from the ITC experiments, two HP1γ-CSD molecules and one TRIM66^856–868^ peptide formed a biological heterotrimer (Fig. 1C). In the final model, most of the residues of HP1γ-CSD protein and all the residues of TRIM66^856–868^ peptide could be unambiguously built (Fig. 1D), with only the electron densities of the N-terminal His_6_ tag, as well as a few C-terminal residues of HP1γ-CSD protein, being invisible probably due to the flexibility. Similar to the apo HP1γ-CSD structure (PDB ID: 3KUP) (supplementary Fig. S1B), each HP1γ monomer folds as an α-β protein composed of a three-stranded antiparallel β sheet (β1–β3) with two amphipathic α helices (α1 and α2) situated on one face of the β sheet. The outside faces of the α2 of two monomers constitute the homodimer interface and form cross-parallel helices bundles, mainly via hydrophobic interactions among several highly conserved residues. Comparison of our structure and the apo HP1γ-CSD structure (all atoms root-mean-square deviation (RMSD) is 0.46 Å) reveals no significant structural changes upon TRIM66^856–868^ peptide binding (supplementary Fig. S1B).

In the structure, the TRIM66^856–868^ peptide adopts an extended conformation and binds on the surface of the HP1γ-CSD homodimer. Although all the residues of the TRIM66^856–868^ peptide could be clearly traced, only a portion of this peptide was found to interact with the HP1γ-CSD protein. The interacting portion of peptide (residues 859–866) is located in the hydrophobic groove between α-helix α1 and the β-sheet, with the other portions of the peptides lying outside the HP1γ-CSD homodimer while making few nonspecific contacts with other neighboring HP1γ-CSD proteins due to crystal packing (Figs. 1C and 1D).

**Figure 2:**
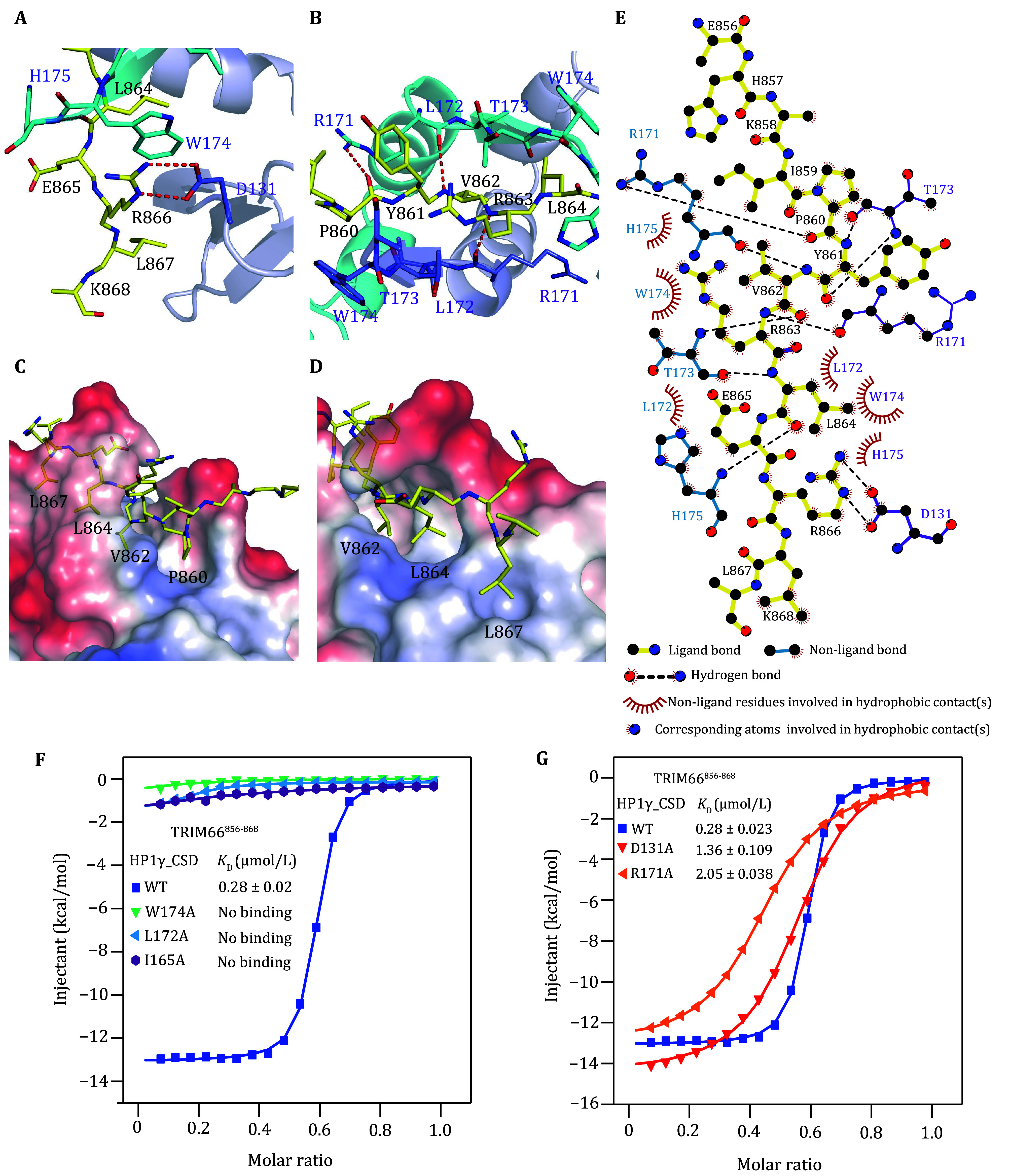
Molecular details between HP1γ-CSD and TRIM66^856–868^. **A**–**D** Close-up views of the interactions of HP1γ-CSD and TRIM66^856–868^ peptide. HP1γ-CSD (grey and cyan) and TRIM66^856–868^ (yellow) are shown as ribbons (**A**, **B**). Hydrogen bonds are shown as red dashed lines. The electrostatic potential of the TRIM66 PxVxL motif peptide complex surface (**C**, **D**), in which positively charged, negatively charged and neutral areas are represented in blue, red and white, respectively. TRIM66^856–868^ peptide is represented as yellow ribbons. **E** Interaction plot between HP1γ-CSD and TRIM66^856–868^ peptide (plot generated with LigPlot^+^ v1.4). **F** ITC fitting curves of HP1γ-CSD^ WT^ (blue squares), HP1γ-CSD^I165A^ (purple circles), HP1γ-CSD^W174A^ (green triangles) or HP1γ-CSD^L172A^ (cyan triangles) with TRIM66^856–868^ peptide. **G** ITC fitting curves of HP1γ-CSD^WT^ (blue squares), HP1γ-CSD^D131A^ (red triangles) or HP1γ-CSD^R171A^ (orange triangles) with TRIM66^856–868^ peptide

In detail, the residues of TRIM66^856–868^ peptide fit into the amphiphilic cavity formed by the hydrophobic residues (L172, W174) and polar residues (R171, T173) of HP1γ-CSD (Figs. 2A–2D). The side chains of the residues P860, V862, L864 and L867 point into the hydrophobic groove of HP1γ-CSD, playing essential roles in HP1γ-CSD-TRIM66 interactions (Figs. 2C–2E). D131 and R171 make a polar network with side chains of residue R866 and the main chain of Y861 of TRIM66^856-868^ peptide (Figs. 2A, 2B and 2E). The importance of the hydrophobic interactions between HP1γ-CSD and TRIM66 was confirmed by mutational analysis. We measured the binding affinities of HP1γ-CSD mutants to wild-type TRIM66^856-868^ peptide. When we mutated L172A and W174A into alanine in HP1γ-CSD, respectively, the affinity between HP1γ-CSD^L172A^ (or HP1γ-CSD^W174A^) and TRIM66^856-868^ peptide was disrupted as indicated by ITC experiments (Fig. 2F and supplementary Table S1). In addition, our mutagenesis studies showed that the mutations D131A and R171A in HP1γ-CSD slightly affected the affinity between HP1γ-CSD and TRIM66^856–868^ peptide (Fig. 2G and supplementary Table S1). To explore whether TRIM66^856–868^ peptide could interact with an HP1γ-CSD without dimer formation. We mutated the key residue located at the homodimer interface (I165A) to disrupt the HP1γ-CSD homodimer. This mutant completely disrupts the homodimer formation. We then performed ITC experiments with wild-type TRIM66^856–868^ peptide titrating into the monomeric HP1γ-CSD mutant. Expectedly, the ITC results showed that TRIM66^856–868^ peptide could not bind to monomeric HP1γ-CSD mutant (Fig. 2F and supplementary Table S1), suggesting a homodimer form of HP1γ-CSD is also essential for its interacting with TRIM66^856–868^.

### TRIM66 interacts with HP1 through its PxVxL motif both *in vitro* and *in vivo*

**Figure 3:**
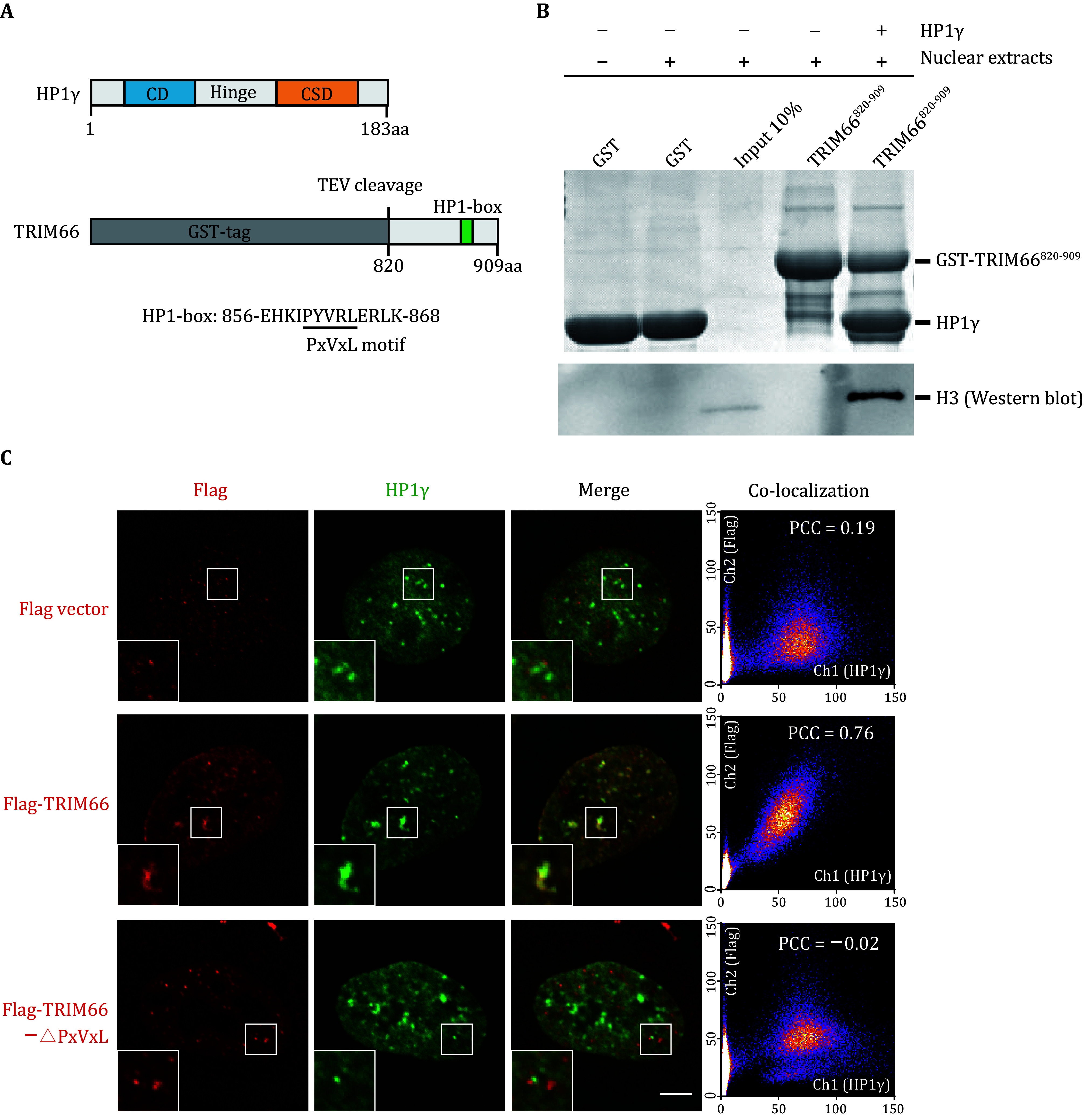
TRIM66 interacts with HP1 through its PxVxL motif. **A** Schematic representation of the domain structures of human HP1γ and TRIM66. In HP1γ, the chromodomain (CD), the chromo shadow domain (CSD), and the hinge region are labeled with different colors. For TRIM66, the N terminal GST tag and the PxVxL motif at the C terminus are highlighted. **B** SDS-PAGE (upper) and western blot (lower) analysis of the pull-down results among GST-TRIM66^820–909^, HP1γ, and nuclear extract (NE). **C** Colocalization and the Pearson coefficient analysis between endogenic HP1γ and overexpressed Flag-tagged wild-type TRIM66 or TRIM66-△PxVxL by Immunofluorescence in fixed U2OS cells. Scale bar, 5 μm

Our previous studies reported that the PHD-Bromo tandem domain of TRIM66 recognizes the unmodified H3R2-H3K4 and acetylated H3K56. However, there is a low affinity between the PHD-Bromo tandem domain of TRIM66 and the H3R2-H3K4-H3K56Ac. The N-terminal Chromo domain (CD) of HP1γ recognizes the H3K9me3 and its C-terminal chromo shadow domain (CSD) interacts with the TRIM66 PxVxL motif. Therefore, we hypothesize HP1γ can assist TRIM66 to interact with nucleosomes. To prove this point, we first explore whether HP1γ, TRIM66 and nucleosomes form complexes *in vitro*. We cloned, expressed and purified full length of HP1γ and GST-TRIM66^820-909^, which contains HP1γ-CSD binding motif (Fig. 3A), and we obtained nuclear extracts (NE) from HeLa cells as described before (Wang *et al.* 2019). The GST pull-down result revealed that GST-TRIM66^820 909^, HP1γ and NE can form stable complexes (Fig. 3B). Next, in order to explore whether TRIM66 and HP1γ are co-localized in eukaryotic cells, we performed immunofluorescence staining with the U2OS cell overexpressed TRIM66 with flag tag. The experimental results and the quantifications show that wild-type TRIM66 and the endogenous HP1γ will form colocalized foci, but when the PxVxL motif of TRIM66 is deleted, this colocalization disappears (Fig. 3C). This result indicates that the PxVxL motif of TRIM66 plays a key role in the interaction between TRIM66 and HP1γ.

### TRIM66 and HP1 phase-separated together in living cells, and TRIM66 is the key regulator of TRIM66-HP1 droplets

**Figure 4:**
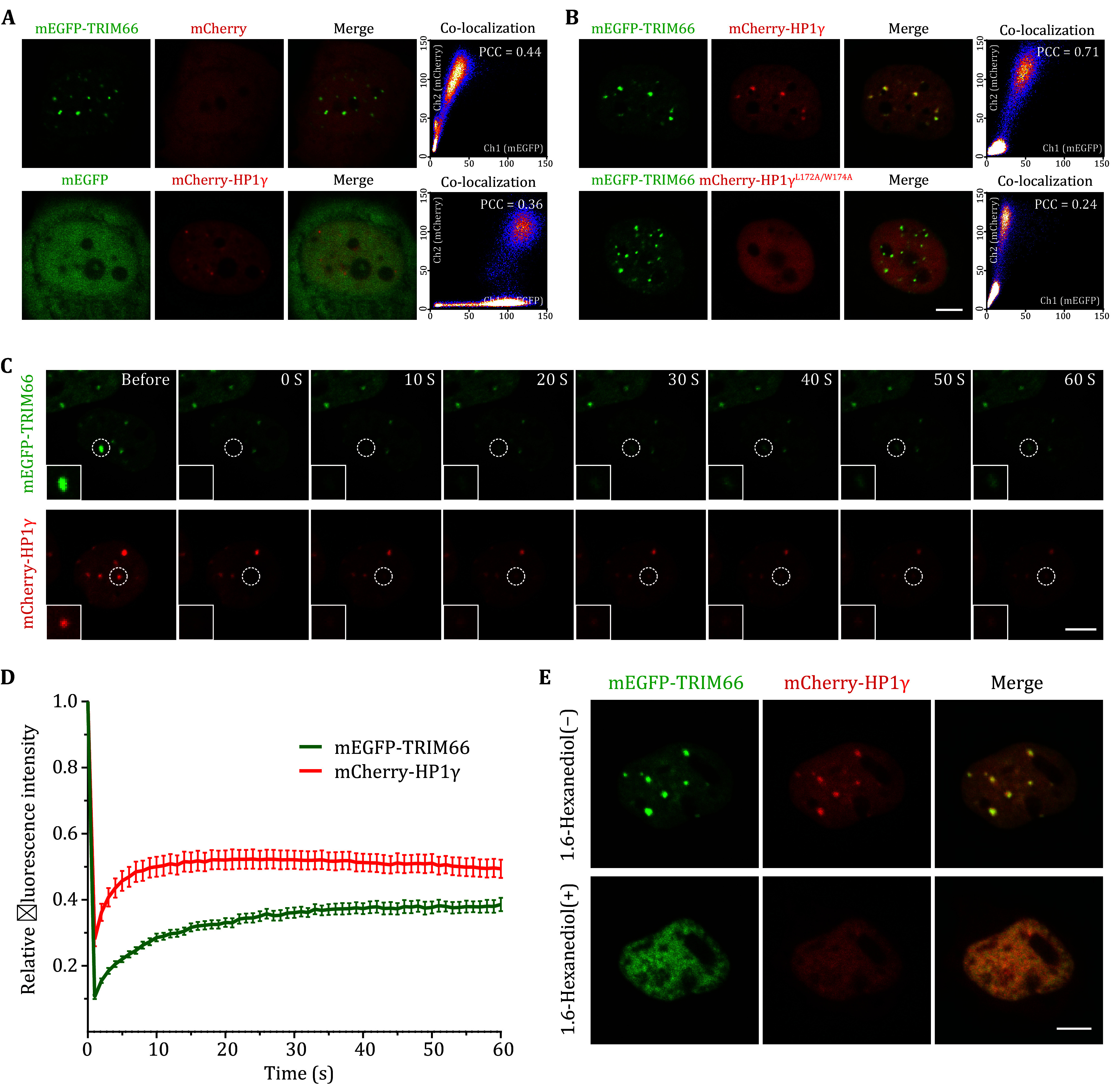
TRIM66 and HP1γ phase separate together in living cells, and TRIM66 is the core for phase separation. **A** Live-cell imaging and the Pearson coefficient analysis of overexpressed mEGFP tagged TRIM66 (Top) and mCherry tagged HP1γ (Bottom) in U2OS cells. Scale bar, 5 μm. **B** Observation and the Pearson coefficient analysis of colocalization between mEGFP tagged TRIM66 and mCherry tagged HP1γ^WT^ (Top) or HP1γ^L172A/W174A^ (Bottom) in U2OS cells. Scale bar, 5 μm. **C** Representative images of mEGFP-TRIM66 and mCherry-HP1γ from the FRAP experiment. Scale bar, 5 μm. **D** Quantification of FRAP data for mEGFP-TRIM66 and mCherry-HP1γ. Data are shown as the mean ± SEM (error bars). *N* = 10 cells per condition. **E** Representative images of mEGFP-TRIM66 and mCherry-HP1γ in one and the same live cell before and after treatment with 10% 1,6-hexanediol for 5 min. 10 independent fields from one cell dish were imaged before and after treatment. Scale bar, 5 μm

To further explore the phase separation ability of TRIM66 and HP1γ, we co-transfected TRIM66 with mEGFP tag and HP1γ with mCherry tag in U2OS cells, as well as the corresponding control vectors, and performed live cell fluorescence observation. The experimental results show that overexpression of mEGFP-TRIM66 alone can form droplet-like condensates, while overexpression of mCherry-HP1γ alone has a weaker ability to form droplets (Fig. 4A). When mEGFP-TRIM66 and mCherry-HP1γ are co-expressed, they can form co-localized droplets, and when the key amino sites where HP1γ bind to TRIM66 is mutated (L174A/W174A), although mEGFP-TRIM66 can still condensate as liquid drops, mCherry-HP1γ is evenly distributed throughout the nucleus (Fig. 4B). Through Fluorescence Recovery Photobleaching (FRAP) experiments, we found that the droplet-like condensates formed by mEGFP-TRIM66 and mCherry-HP1γ are highly mobile in the living cell (Figs. 4C and 4D). Moreover, the condensates formed by mEGFP-TRIM66 and mCherry-HP1γ were depolymerized and the fluorescence signal was evenly distributed in the entire nucleus when 1,6-hexanediol, which can disrupt hydrophobic interaction between proteins, was added to the cell culture medium (Fig. 4E). The above experimental results show that the TRIM66-HP1γ droplet-like condensates in the nucleus is a liquid-liquid phase separation phenomenon, and TRIM66 is the core driver for the formation of the TRIM66-HP1 phase separation droplets.

### TRIM66 BBCC domain is the core that mediates the phase separation

**Figure 5:**
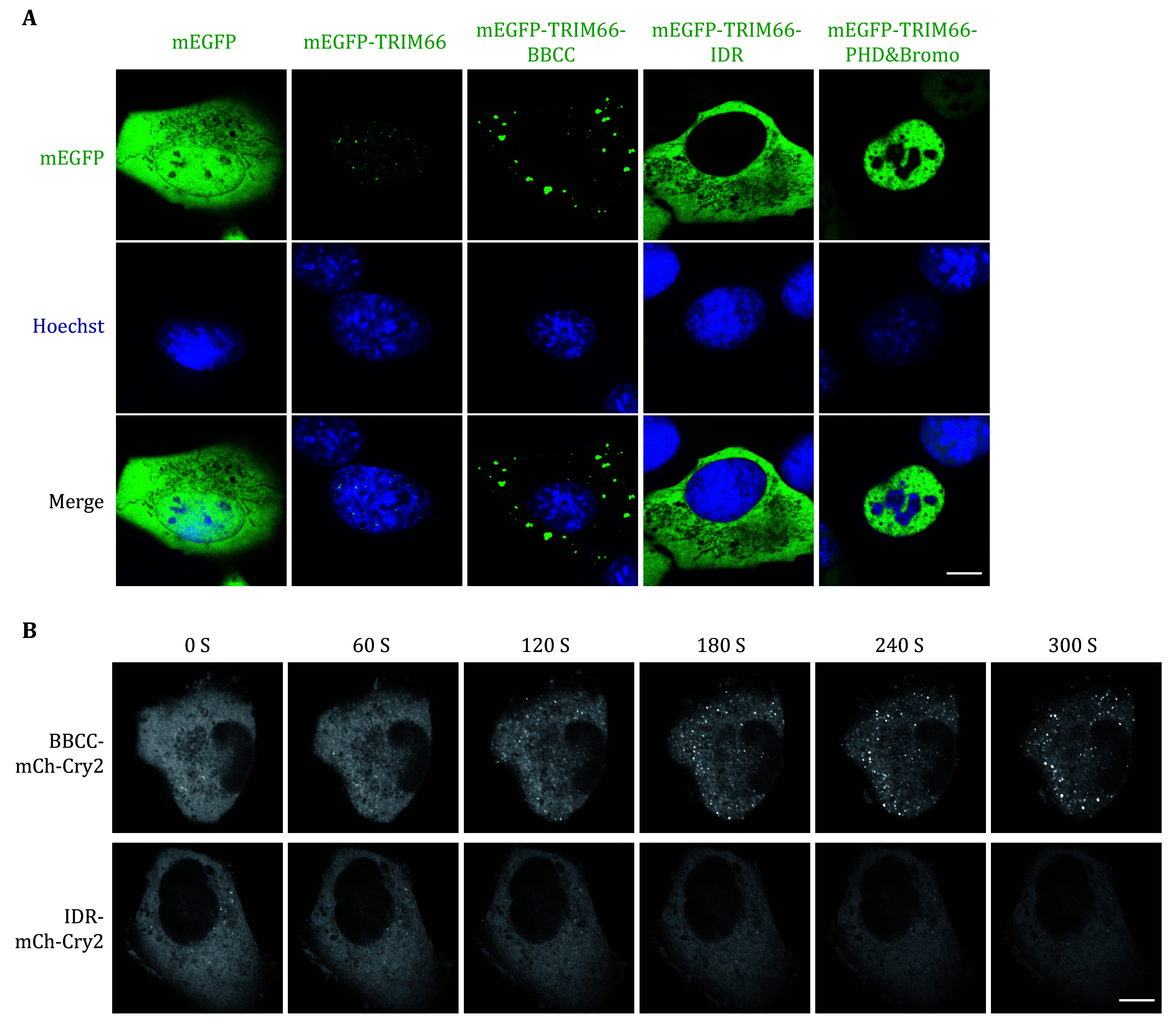
BBCC domain is the core domain of TRIM66 that mediates phase separation. **A** U2OS cells were transfected with different mEGFP-TRIM66 constructs and the nuclei were stained with Hoechst-33342. Scale bar, 10 μm. **B** An opto-droplet assay. Cells expressing two TRIM66 constructs with mCherry and Cry2 linked on the C terminus tandemly were induced by blue light followed by time-lapse imaging. Scale bar, 10 μm

To further explore the key domains where TRIM66 mediates phase separation occurs, we truncated TRIM66 and transfected different fragments with mEGFP tag into U2OS cells for phase separation capability detection. Experimental results showed that only BBCC of TRIM66 can produce droplet condensates, while IDR or PHD & Bromo domains do not when overexpressed, and the overexpressed BBCC and IDR domain alone are mainly located in the cytoplasm, which indicates that PHD & Bromo domain mediates the nucleus localization of TRIM66 (Fig. 5A and supplementary Fig. S2B). We further confirmed the ability of BBCC to induce phase separation by domain deletion experiments. When BBCC is removed from the full-length TRIM66 protein (△BBCC), the localization of TRIM66 in the nucleus becomes relatively diffuse, but there are still some condensates. We speculate that this may be caused by the localization to the heterochromatin region due to the endogenous proteins. When the PxVxL motif is deleted from the full-length TRIM66 protein (△PxVxL), TRIM66 forms a more significant droplet-like condensate in the nucleus. Correspondingly, when both the PxVxL motif and BBCC domains are deleted at the same time (△BBCC&PxVxL), TRIM66 is distributed in the nucleus, and no droplet aggregation occurs (supplementary Fig. S2). Through the light-induced phase separation system (Opto-droplet/CRY2 system), we further confirmed the phase separation ability of BBCC (Shin *et al.* 2017; Taslimi *et al.* 2014). When BBCC or IDR of TRIM66 were fused with mCherry and CRY2, respectively, only the fusion protein of BBCC could form droplet condensates under the stimulation of blue light (Fig. 5B).

### HP1γ-TRIM66δ forms puncta in the presence of nuclear extract *in vitro*

**Figure 6:**
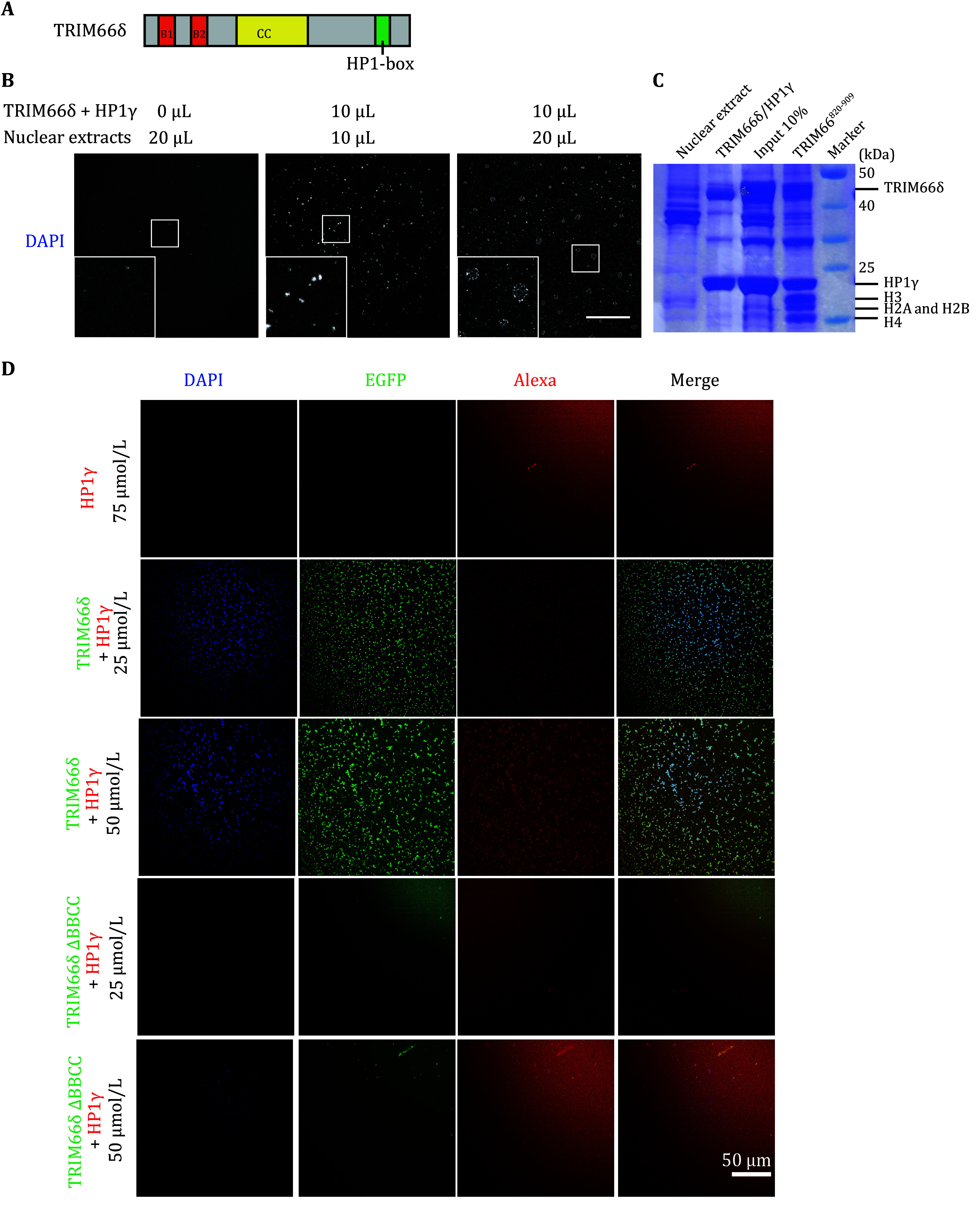
LLPS assay of human HP1γ, TRIM66 and nuclear extracts (NEs) *in vitro*. **A** Schematic representation of TRIM66δ. In TRIM66δ, the B-box motif (B1), the B-box motif 2 (B2), the coiled-coil region (CC) and HP1-box are colored as above. **B** LLPS assay of human HP1γ, TRIM66 and NEs *in vitro*. Images were acquired at room temperature. All protein concentrations were at 100 μmol/L. Scale bar, 50 μm. **C** SDS-PAGE analysis of NEs, HP1γ, TRIM66 and fractions from phase separation. **D** Phase separation assay of NE-HP1γ with different TRIM66 constructs. Scale bar, 50 μm

To investigate the contribution of TRIM66 to focus formation by LLPS, we used the *in vitro* system containing HP1γ, TRIM66δ (a construct of TRIM66, containing BBCC and PxVxL motif), and Nuclear Extracts (Nes). By running the SEC-MALS (Size Exclusion Chromatography coupled with Multi-Angle static Light Scattering) assay, we noticed a significantly increased mass of TRIM66δ-HP1γ complex than the TRIM66δ alone (supplementary Figs. S3A and S3B). We next carried out binding reactions by mixing NE strained by DAPI with TRIM66δ-HP1γ or only with HP1γ. We found that NE mixing with TRIM66δ-HP1γ can observe puncta clearly and the SDS-PAGE analysis showed that there are TRIM66δ, HP1γ and NE containing four prominent histone bands in the puncta (Figs. 6A–6C). While the FRAP experiment also demonstrated the fluidity of the foci containing TRIM66δ, HP1γ, and NE (supplementary Figs. S3C and S3D). These results proved that TRIM66 and HP1γ play important roles in foci formation *in vitro*. Further, we want to prove BBCC is the key domain for TRIM66-inducing phase separation *in vitro*. To label HP1γ with a fluorescent probe distinct from that labeling TRIM66δ, we produced Alexa 568 stained HP1γ and EGFP-TRIM66δ and EGFP-TRIM66δ△BBCC (Fig. 6D). Consistent with the result in the cell, we found that EGFP-TRIM66δ△BBCC loss ability inducing phase separation *in vitro*.

### HP1γ induces TRIM66 phase separation in the heterochromatin region and inhibits gene expression

Next, we explored the relationship between the phase separation of TRIM66 with HP1γ and the formation of heterochromatin. Consistent with previous reports, by immunofluorescence staining, we found that HP1γ co-localized with the heterochromatin region marker H3K9me3 (supplementary Fig. S4A). At the same time, overexpressed wild-type FLAG-tagged TRIM66 was also colocalized with H3K9me3. However, when the PxVxL motif of TRIM66 was deleted, this colocalization disappeared (supplementary Fig. S4B). The results indicate that HP1γ is the core protein that mediates co-phase separation system localization in the heterochromatin region.

**Figure 7:**
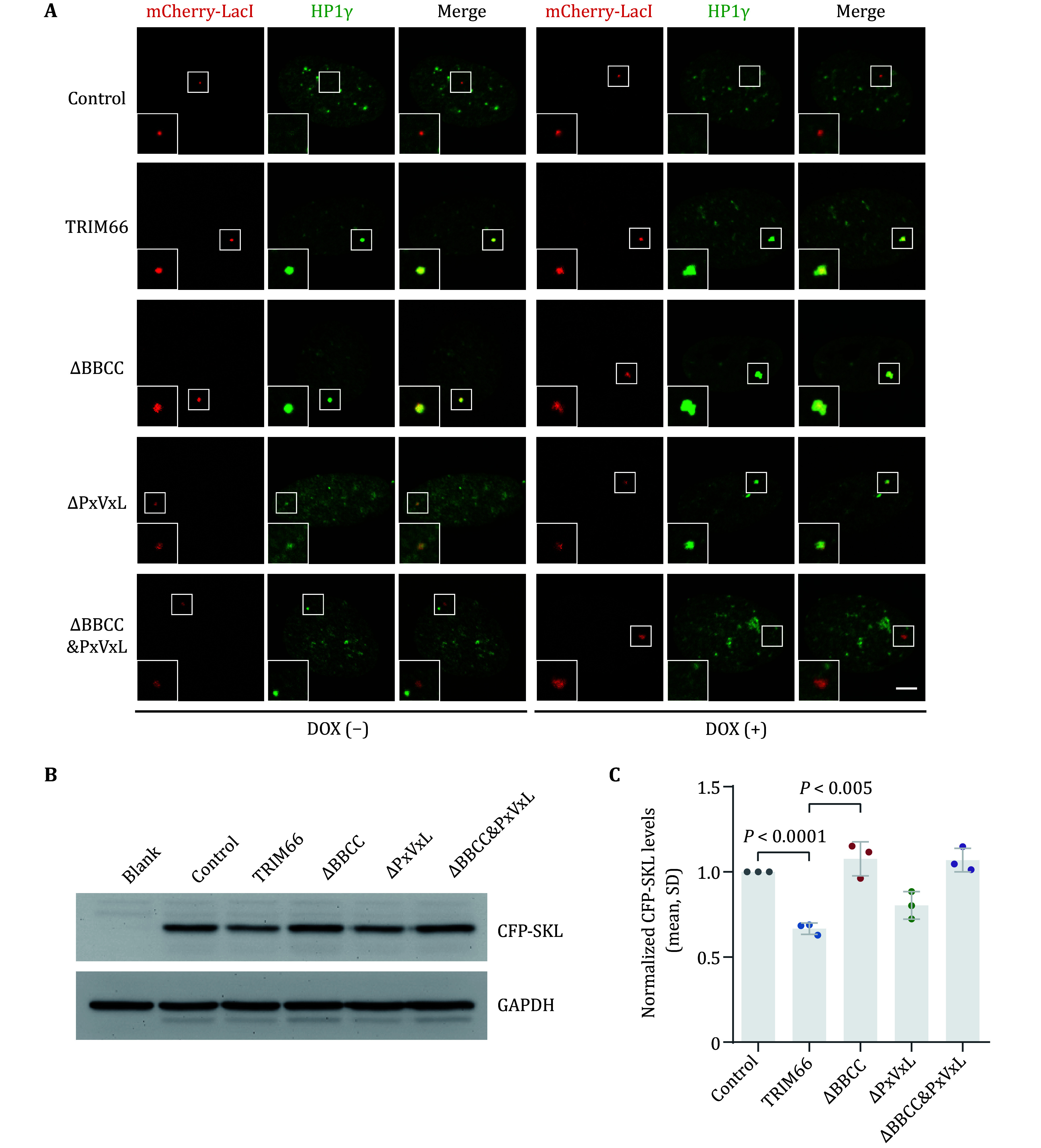
HP1γ-TRIM66 complex induces phase separation in heterochromatin region and inhibits gene expression. **A** Colocalization between TRIM66 or its various truncations fused with mCherry-LacI and endogenic HP1γ by Immunofluorescence in fixed U2OS cells. Cells were treated with 1 μg/mL DOX for 3 hours before fixed. Scale bar, 5 μm. **B** Western blot analysis of the effect of overexpression of different fusion proteins on the expression of reporter gene CFP-SKL by α-GFP antibody. **C** Gray value analysis of western blotting images. Data from 3 independent experiments

In order to explore the effect of co-phase separation of TRIM66-HP1γ on gene transcription, we introduced a gene transcription reporter system. The cell line U2OS 2-6-3, into which multiple copies of the Lac operator (Lac O) repeat and a CFP reporter gene whose expression can be induced by doxycycline (Dox) had been stably integrated into a euchromatic region of chromosome 1, was constructed by Susan M. Janicki *et al.* as previously reported (Janicki *et al.* 2004). We expressed TRIM66 or its various truncations in fusion with mCherry-LacI in this cell line and induced the expression of the reporter gene CFP-SKL by adding doxycycline (Dox). By simultaneously staining endogenous HP1γ by immunofluorescence, we found that before and after treated with DOX, mCherry-LacI alone in the control group and the fused protein of mCherry-LacI and TRIM66-△BBCC&PxVxL in the experimental group did not co-localized with HP1γ, but fusion proteins of mCherry-LacI with full-length TRIM66, TRIM66-△BBCC or TRIM66-△PxVxL always co-localized with HP1γ (Fig. 7A). Still, the puncta’s patterns were different due to the functions of various regions. Further, we detected the expression of reporter gene CFP-SKL under the condition of transfection of different fusion proteins by Western-blot experiment. The experimental results showed that after adding Dox to induce expression for the same time, the expression of CFP was significantly reduced only when the fusion protein of mCherry-LacI and the full-length or truncated TRIM66 containing the BBCC was overexpressed (Figs. 7B and 7C).

## DISCUSSION

Cells form different membrane-bound organelles to separate different compartments and operate different functions, like mitochondria, which mainly produce energy, and the nucleus, which stores the chromatin. Besides these, the cell also contains lots of different types of membraneless organelles (MLO). The MLO is formed through liquid-liquid phase separation and driven by weak multivalent interactions. There are lots of reports about the MLO induced by the intrinsically disordered region in a protein or the nucleic acids binding protein and the corresponding DNA or RNA. But here we reported that the TRIM66 formed a liquid granule through its BBCC fragment, which contained tandem two B-box domains and coiled-coil domain, to assist the HP1γ in remodeling the facultative heterochromatin. Besides the HP1γ, the other two heterochromatin proteins 1 (HP1) also reshape the chromatin regions containing the H3K9me3 modification through LLPS (HP1α itself or HP1β with the help of TRIM28). So, our work described how HP1γ, the only HP1 family that doesn’t understand the phase separation behavior, rebuilds the facultative heterochromatin through the liquid-liquid phase separation.

**Figure 8:**
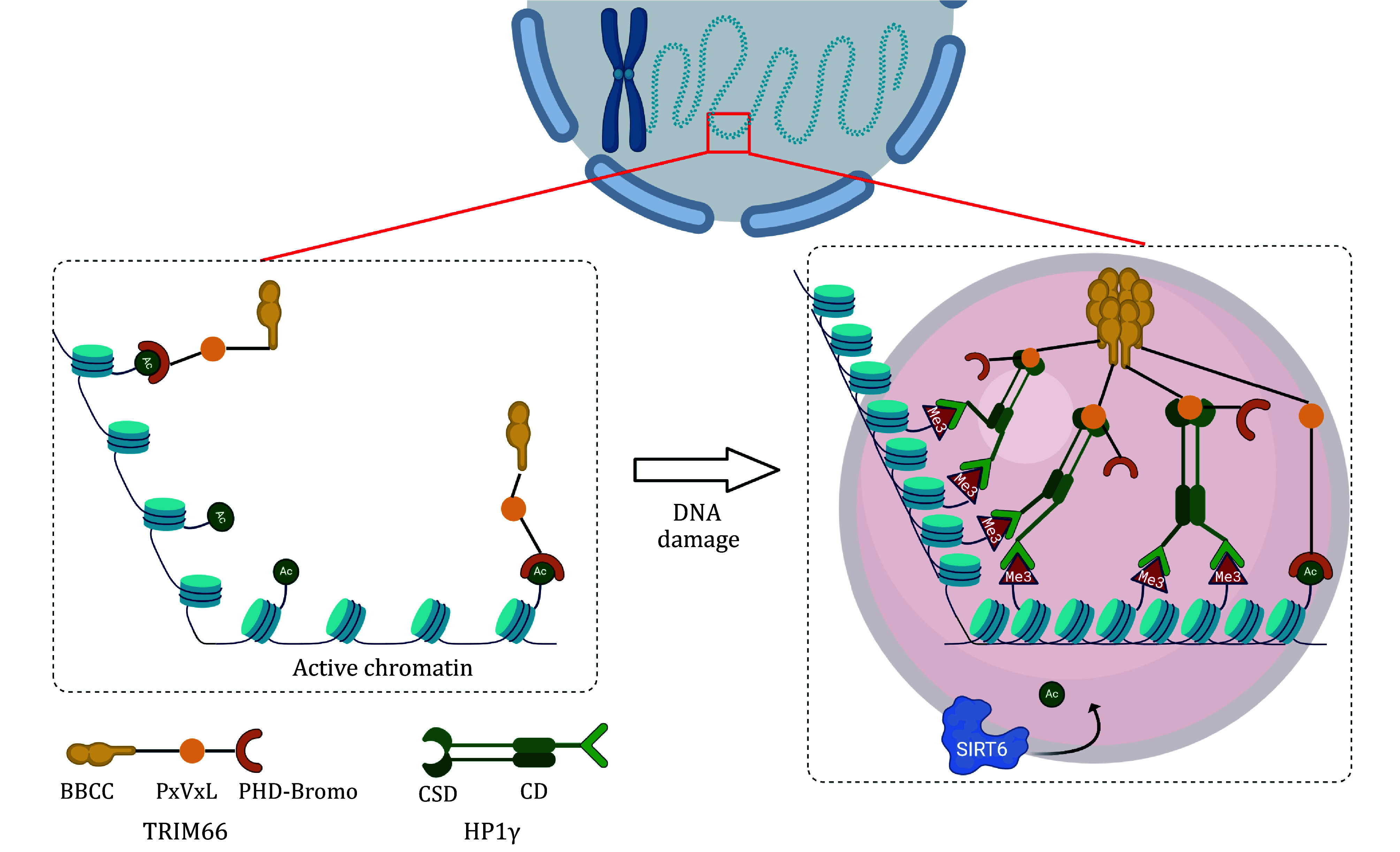
Model of chromatin condensation formation driven by TRIM66-HP1γ-H3K9me3 axis (The model was generated from BioRender.com)

Combined with our previous research (Chen *et al.* 2019), we demonstrated the mechanism of how TRIM66 responds to DNA damage (Fig. 8): TRIM66 recognized the unmodified H3R2-H3K4 and acetylated H3K56, which marks the active chromatic regions, through its PHD and Bromo domains during the regular period. When DNA damage happens, DNA damage evokes a serial DNA coordinating responding pathway, which will condense the chromatin and suppress the gene expression (Fortuny *et al.* 2021). There are reports suggesting that the trimethylation of histone H3 lysine 9 (H3K9me3) labeled the barrier of the double-strand breaks area (DSB) (Young *et al.* 2013). Around the H3K9me3 markers, HP1γ was recruited to recognize the H3K9me3 by its CD domain. TRIM66 was subsequently gathered into the DSB region by interactions between the PxVxL motif of the TRIM66 and the CSD domain of HP1γ. Then the BBCC domain of TRIM66 drove the marked chromatin to rebuild into the facultative heterochromatin to “lock” the damaged range and wait for the DSB repair machine through the liquid–liquid phase separation mechanism. Later on, the TRIM66 reads the unmodified H3R2K4 and H3K56ac with C-terminal PHD and Bromo domains and recruits the SIRT6 to deacetylate H3K56ac, down-regulate the H3K56ac level and initiate the assembly of the DNA damage repair engine to maintain the genomic stability.

## METHODS AND MATERIALS

### Protein expression and purification

HP1γ_CSD (residues 110–176) was amplified by PCR from the human brain cDNA library and cloned into the pET-28a(+) expression vector (Novagen), contained an N-terminal 6× His tag and a tobacco etch virus (TEV) protease cleavage site. All mutants of HP1 were generated using a MutantBEST kit (Takara) and confirmed by DNA sequencing. All the proteins were expressed in *Escherichia coli* BL21 (DE3) cells (Novagen) cultured in LB medium at 37 °C to OD_600_ = 0.8, then shifted to 16 °C and induced with 0.5 mmol/L isopropyl β-D-1-thiogakactopyranoside (IPTG) for 24 h. Bacterial pellets were resuspended in Buffer A (20 mmol/L Tris-HCl, 500 mmol/L NaCl, pH 7.5) and lysed by sonication on ice. Then, soluble proteins were purified with a Ni^2+^-chelating column (Cytiva Life Sciences) and a Superdex 75 column (Cytiva Life Sciences). After being cleaved by TEV protease overnight at 4 °C to remove the 6× His tag, the purified protein was concentrated to ∼10 mg/mL in Buffer B (20 mmol/L Tris-HCl, 150 mmol/L NaCl, pH 7.5) and stored at –80 °C.

The gene for human TRIM66 full-length (residues 1–1050) was purchased from Life Technologies. All the DNA fragments for the human TRIM66 (residues 820–909, 840–909) were amplified by PCR. The proteins were cloned into a modified pGEX4T-1vector with a GST tag and TEV protease cleavage site. The proteins were expressed in *Escherichia coli* BL21 (DE3) cells and induced with 0.5 mmol/L IPTG for 24 h at 16 °C. The protein was purified by GST column (Cytiva Life Sciences), followed by TEV protease cleavage and size-exclusion chromatography on a Hiload 16/60 Superdex 200 column (Cytiva Life Sciences).

### Preparation of peptide

Peptide TRIM66 PxVxL motif (residues 856–868) used in the study were chemically synthesized by GL Biochem (Shanghai, China) and dissolved in Buffer B (20 mmol/L Tris-HCl, 150 mmol/L NaCl, pH 7.5) a final concentration of 10 mmol/L.

### Isothermal titration calorimetry

Isothermal titration calorimetry (ITC) assays were performed using a MicroCal PEAQ-ITC calorimeter (Cytiva Life Sciences), and all experiments were carried out at 20 °C. Buffer B was used for sample dilution. The concentrations of the proteins were determined spectrophotometrically. Data analysis was done using MicroCal PEAQ-ITC Analysis Software provided by the manufacturer. The thermodynamic parameters of the ITC experiments are listed in supplementary Table S1.

### Crystallization, data collection and structure determination

HP1γ CSD (110–176aa) and TRIM66 PxVxL motif peptide(856–868aa), mixed at a molar ratio of 1:1.2, were crystallized in 30% PEG MME 2000, 0.1 mmol/L sodium cacodylate (pH 6.0) at 16 °C by vapor diffusion in sitting drops. Crystals were soaked in cryo-protectants made of mother liquor supplemented with 25% glycerol before being flash-frozen in liquid nitrogen. Data sets were collected on Beamline 19U at the Shanghai Synchrotron Radiation Facility (SSRF). The structure of the HP1γ CSD and TRIM66 PxVxL motif complex was solved by molecular replacement with the program MOLREP (Vagin and Teplyakov 2010), using apo HP1β 108–185 (PDB ID: 3Q6S) as the search model (Kang *et al.* 2011). The TRIM66 PxVxL motif peptide (856–868 aa) peptide was then modeled in COOT (Emsley *et al.* 2010), and the structure of the HP1γ CSD-TRIM66 PxVxL motif complex was refined by the programs REFMAC5 (Winn *et al.* 2011) and PHENIX.refine (Adams *et al.* 2010). Experimental structure factors and the coordinates of the final model have been deposited into the Protein Data Bank (PDB) with access code 8JZW. Crystal diffraction data and refinement statistics are summarized in supplementary Table S2.

### Preparation of nuclear extracts

Nuclear extracts (NEs) were prepared as described with slight modifications. HeLa cells (1 × 10^8^ cells/mL) were suspended with 4 mL of Buffer A comprising 0.5 mmol/L EDTA, 2 mmol/L MgCl_2_, 0.1% (*v*/*v*) protease inhibitor (PI) (Nakalai Tesque, Japan), 2 mmol/L β-mercaptoethanol, 10 mmol/L Tris–HCl, pH 7.8, and centrifuged at 1500 *g* for 2 min at 4 °C. Pellet was suspended with 4 mL of Buffer A containing 0.025% (*w*/*v*) Triton-X100. The suspension was centrifuged at 1500 *g* for 2 min at 4 °C, and then the pellet was washed twice with 4 mL of Buffer A. The precipitate was re-suspended with 0.75 mL of buffer comprising 1 mmol/L KCl, 1 mmol/L CaCl_2_, 0.1% (*v*/*v*) PI, 0.34 mol/Lsucrose, 10 mmol/L Tris–HCl, pH7.4, and then treated with 4.3 U/mL of micrococcal nuclease at 37 °C for 15 min to prepare oligo-nucleosomes. The reaction was terminated by adding a final concentration of 5 mmol/L EDTA and then the mixture was centrifuged at 2000 *g* for 2 min at 4 °C. The precipitate was then suspended with 0.7 mL buffer comprising 1 mmol/L EDTA, 0.1% (*v*/*v*) PI, 10 mmol/L Tris–HCl, pH 7.4, and then centrifuged at 2000 *g* for 2 min at 4 °C. The supernatant fraction was recovered as oligo-nucleosomes. Oligo-nucleosomes thus prepared were dialyzed against 0.1% (*v*/*v*) PI and 10 mmol/L Tris–HCl, pH 7.4, at 4 °C. The concentrations of NEs were determined by DNA concentrations calculated from the absorbance at 260 nm.

### GST pull-down assays

The GST alone control, GST-tagged protein (TRIM66^820–909^) was bound to glutathione-Sepharose beads (Cytiva Life Sciences) in a buffer of 20 mmol/L Tris, 200 mmol/L NaCl, and 1 mmol/L DTT at pH 7.5, and incubated at 4 °C for 4 h. The glutathione-sepharose beads were then spined down and washed three times with the washing buffer. Then the beads were incubated with recombinant HP1γ and NE in a buffer including 20 mmol/L Tris, 150 mmol/L NaCl and 1 mmol/L DTT at pH 7.5 for 6 h at 4 °C. The beads were subsequently pelleted and washed three times using the same buffer. The proteins captured by beads were eluted and analyzed by SDS-PAGE and western blot.

### Cell culture and transfection

U2OS cells and HeLa cells were cultured in high-glucose DMEM supplemented with 10% fetal bovine serum and 1% penicillin-streptomycin at 37 °C in a humidified incubator containing 5% CO_2_. Following the manufacturer's instructions, cells were transfected with plasmid DNA using Lipofectamine 3000 (Thermo Fisher Scientific).

### Plasmids and antibodies

Genes encoding human TRIM66 or its constructs were cloned into the pmEGFP-C3 (Clontech) vector with an N-terminal mEGFP tag or the Flag vector with an N-terminal Flag tag. Genes encoding human HP1γ or HP1γ^L172A/W174A^ were cloned into the pcDNA3.1 vector with an N-terminal mCherry tag.

HP1γ (ab217999, 1:1000 dilution for IF) and H3K9me3 (ab8898, 1:500 dilution for IF) antibodies were purchased from Abcam. Flag (F1804, 1:1000 dilution for IF) antibody was purchased from Sigma-Aldrich. GFP (50430-2-AP, 1:2000 dilution for WB) and GAPDH (60004-1-Ig, 1:10000 dilution for WB) antibodies were purchased from Proteintech.

### Live-cell imaging and data processing

U2OS cells were cultured on 35-mm glass bottom dishes (Wuxi NEST Biotechnology Co., Ltd) coated with poly-D-lysine (Sigma-Aldrich). L-15 medium without phenol red (Thermo Fisher Scientific) was used during live-cell imaging to maintain a suitable environment for cell growth, and the temperature was kept at approximately 37 °C. Images were obtained with a Nikon Ti fluorescent inverted microscope with a 100× 1.4 NA objective; an XY Piezo Z stage (Applied Scientific Instrumentation); a spinning disk confocal unit (Yokogawa); and an electron multiplier CCD camera (Andor) controlled by IQ3 live-cell imaging software (Andor). Images collected with this system were 16-bit and consisted of 512 × 512 pixels, and the pixel size was 0.12 μm.

### Fluorescence recovery after photobleaching

The Fluorescence recovery after photobleaching (FRAP) experiment was performed with a Nikon A1R HD25 confocal microscope. mEGFP-TRIM66 were excited by a 488 nm laser, and 40% laser power was used for bleaching. mCherry-HP1γ were excited by a 561 nm laser, and 60% laser power was used for bleaching. Images were captured every 1 s with a 60× 1.49 NA objective after bleaching. The fluorescence intensity at the bleached spot was measured with the FIJI plugin FRAP Profiler V2.

### Immunofluorescence (IF)

U2OS cells were cultured on 18 mm × 18 mm glass coverslips coated with poly-D-lysine (Sigma-Aldrich) at the bottom of 35-mm dishes (Wuxi NEST Biotechnology Co., Ltd). After 48 h of gene transfection, the cells were fixed in 4% paraformaldehyde for 10 min, followed by permeabilization with PBS buffer containing 0.4% Triton X-100 for 10 min. After that, the cells were blocked with PBS buffer containing 5% BSA for 30 min, then incubated sequentially with primary and secondary antibodies.

## Conflict of interest

Siyuan Shen, Feng Chen, Yifan Zhang, Fudong Li, Xuebiao Yao, Dan Liu, Yunyu Shi and Liang Zhang declare that they have no conflict of interest.
